# Emotional behavior and brain anatomy of the *mdx52* mouse model of Duchenne muscular dystrophy

**DOI:** 10.1242/dmm.049028

**Published:** 2021-09-21

**Authors:** Amel Saoudi, Faouzi Zarrouki, Catherine Sebrié, Charlotte Izabelle, Aurélie Goyenvalle, Cyrille Vaillend

**Affiliations:** 1 Université Paris-Saclay, CNRS, Institut des Neurosciences Paris Saclay, 91190, Gif-sur-Yvette, France; 2 Université Paris-Saclay, UVSQ, Inserm, END-ICAP, 78000 Versailles, France; 3 Université Paris-Saclay, CEA, CNRS, Inserm, BioMaps, Service Hospitalier Frédéric Joliot, 4 place du général Leclerc, 91401 Orsay, France

**Keywords:** Duchenne muscular dystrophy, Brain dystrophins, DMD mouse model, Fear conditioning, Anxiety, Intellectual disability

## Abstract

The exon-52-deleted *mdx52* mouse is a critical model of Duchenne muscular dystrophy (DMD), as it features a deletion in a hotspot region of the *DMD* gene, frequently mutated in patients. Deletion of exon 52 impedes expression of several brain dystrophins (Dp427, Dp260 and Dp140), thus providing a key model for studying the cognitive impairment associated with DMD and testing rescuing strategies. Here, using *in vivo* magnetic resonance imaging and neurohistology, we found no gross brain abnormalities in *mdx52* mice, suggesting that the neural dysfunctions in this model are likely at the level of brain cellular functionalities. Then, we investigated emotional behavior and fear learning performance of *mdx52* mice compared to *mdx* mice that only lack Dp427 to focus on behavioral phenotypes that could be used in future comparative preclinical studies. *mdx52* mice displayed enhanced anxiety and a severe impairment in learning an amygdala-dependent Pavlovian association. These replicable behavioral outcome measures are reminiscent of the internalizing problems reported in a quarter of DMD patients, and will be useful for preclinical estimation of the efficacy of treatments targeting brain dysfunctions in DMD.

## Abstract (precis)

**Summary:** The *mdx52* mouse model of Duchenne muscular dystrophy lacks brain Dp427 and Dp140 dystrophins, and exhibits emotional, behavioral and learning phenotypes that are relevant for use in preclinical studies of the disorder.

## INTRODUCTION

X-linked Duchenne muscular dystrophy (DMD) is a recessive neuromuscular syndrome that affects one out of 3500 to 5000 male births. Besides the well-characterized muscular dystrophy, DMD is frequently associated with non-progressive cognitive deficits and comorbid behavioral and neuropsychiatric disturbances (Colombo et al., 2017; Hinton et al., 2009; Ricotti et al., 2016). DMD occurs as a result of mutations in the *DMD* gene, composed of 79 exons with multiple independent internal promoters that control the expression of different dystrophin-gene products, known as dystrophin proteins (Dp), in a tissue and/or cell-specific manner. The distinct dystrophins thus differ by their molecular weight, their tissue and cellular distribution, and their roles, but have the common property of being associated with scaffolds of transmembrane and cytosolic proteins involved in the clustering of various membrane ion channels and receptors. The full-length dystrophin Dp427 (427 kDa) is expressed in both muscle and the central nervous system (CNS), Dp260 (260 kDa) is only detected in the retina, and Dp140 (140 kDa) and Dp71 (71 kDa) are detected in the brain. Therefore, the position of a mutation or deletion within the *DMD* gene may lead to the loss of different brain dystrophins (Hoffman et al., 1987), hence complex phenotype-genotype relationships and various degrees of cognitive and behavioral disturbances. Proximal mutations that selectively affect the expression of Dp427 are associated with mild central alterations, whereas distal mutations causing a cumulative loss of Dp427 and loss of one or both of the shorter brain dystrophins Dp140 and Dp71 are frequently linked to severe cognitive deficits and lower IQ scores (intellectual disability) (Daoud et al., 2009a; Felisari et al., 2000; Lorusso et al., 2013; Ricotti et al., 2016; Taylor et al., 2010).

Structure-function and functional studies of different mouse models of DMD may provide an essential contribution to our understanding of the affected brain mechanisms depending on the position of the mutation and loss of different dystrophins. The original Dp427-deficient *mdx* mouse model of DMD features a nonsense mutation in exon 23 of the gene, leading to the loss of both muscle and brain Dp427. The *mdx* mouse has been extensively studied, and it was shown that the loss of brain Dp427 alters brain GABAergic inhibition, and is associated with emotional disturbances and mild cognitive deficits (Knuesel et al., 1999; Vaillend et al., 1995, 2004; Vaillend and Chaussenot, 2017a). The loss of brain Dp427 appears to particularly alter the functionality of the neuronal network involved in the processing of fear responses, as demonstrated in *mdx* mice by the presence of drastically enhanced fearfulness in response to mild stress and delayed fear learning and memory (Goyenvalle et al., 2015; Razzoli et al., 2020; Sekiguchi et al., 2009; Vaillend and Chaussenot, 2017). In contrast, the selective loss of Dp71 in mice does not induce muscular dystrophy (Sarig et al., 1999) but alters critical molecular mechanisms involved in excitatory synapse and astrocyte functions (Belmaati Cherkaoui et al., 2020; Daoud et al., 2009b), which results in a different profile of behavioral disturbances, including deficits in spatial learning, working memory and cognitive flexibility (Chaussenot et al., 2019; Helleringer et al., 2018). However, so far, there has been very limited behavioral studies of mouse models with distal mutations leading to cumulative loss of several dystrophins (Vaillend et al., 1998; Vaillend and Ungerer, 1999). In particular, no behavioral studies have been conducted in mice featuring a mutation in the ‘hotspot’ central region of the *Dmd* gene, which is frequently mutated in DMD patients (63%) and leads to a cumulative loss of Dp427 and Dp140, despite its association with more severe cognitive impairments (Ricotti et al., 2016; Felisari et al., 2000; Beroud et al., 2007; Daoud et al., 2009a; Taylor et al., 2010; Lorusso et al., 2013; Colombo et al., 2017).

A mouse model with a deletion in exon 52 of the *Dmd* gene, the *mdx52* mouse (Araki et al., 1997), is an interesting model for preclinical studies addressing both muscular and CNS dysfunctions, as it is deficient in both Dp427 and Dp140. Although *mdx52* mice display clear and typical muscular dysfunctions, their behavioral disturbances need to be characterized. The *mdx52* mice are expected to display at least comparable cognitive and behavioral deficits as the ones observed in the original *mdx* mouse lacking Dp427. However, the impact of the additional loss of Dp140 in *mdx52* mice is unknown and might influence the severity and/or nature of the central deficits. The cellular function of Dp140 is largely unknown (Lidov et al., 1995) but its higher expression in fetal brain compared to adult brain (Morris et al., 1995) suggests that its loss might affect fetal brain formation and development. Importantly, it has been shown that exon-skipping strategies based on intracerebral or systemic administration of antisense sequences are efficient at skipping exon 23 to restore the reading frame and partially re-express Dp427 in the brain of *mdx* mice, thereby improving GABAergic functions, synaptic plasticity and behavioral fear responses in this model (Dallérac et al., 2011; Goyenvalle et al., 2015; Vaillend et al., 2010). Recent studies demonstrated that the *mdx52* mouse is also eligible for exon-skipping strategies using systemic administration of naked or vectorized antisense sequences to skip exon 51, with therapeutic potential to restore muscle Dp427 expression and motor functions (Aoki et al., 2010; Aupy et al., 2020). Therefore, it is important to determine the behavioral profile and neurobiological bases of central deficits of *mdx52* mice to identify the relevant outcome measures for using this model in preclinical studies.

In the present study, we have undertaken a behavioral characterization of the *mdx52* mouse compared to the original *mdx* mice and respective wild-type littermate controls in order to estimate the presence and severity of the deficits, with a focus on the main phenotypes previously described and used in preclinical studies in the original *mdx* mouse. We also performed a first characterization of brain anatomy, to determine whether Dp140 loss induced gross malformations that were not detected in previous studies of the *mdx* mouse, by means of macroscopic volumetric measures using *in vivo* magnetic resonance imaging (MRI) and analysis of brain architectural organization using histological staining of brain sections. We show that *mdx52* mice display emotional disturbances and a deficit in fear learning that are more severe than in the original *mdx* mouse lacking only Dp427. No gross brain abnormalities were detected in *mdx52* mice, indicating that specific cellular and physiological dysfunctions should be further investigated to determine the neural basis of brain dysfunctions in this new model of DMD.

## RESULTS

### Neuroanatomical analyses

The lack of exon 52 in the *Dmd* gene in *mdx52* mice results in the lost expression of several dystrophins proteins in the CNS, i.e. Dp427, Dp260 and Dp140, whereas expression of the shorter Dp71 product is preserved, as previously shown by western blot analyses of retinal and brain samples in the original biochemical characterization of this mouse model (Araki et al., 1997). Using immunofluorescence and confocal image analyses of hippocampal and cerebellar cryosections, we confirmed that *mdx52* mice lack the full-length brain dystrophin (Dp427), which normally shows punctate immunoreactive signals in wild-type mice due to its expression in brain inhibitory synapses (Fig. S1A). We also confirmed the preserved expression of Dp71, typically detected along the walls of blood vessels in both wild-type and *mdx52* mice (Fig. S1B), due to its main expression at the membrane of perivascular astrocyte endfeet forming the blood-brain barrier (Belmaati Cherkaoui et al., 2020).

**Fig. 1.**
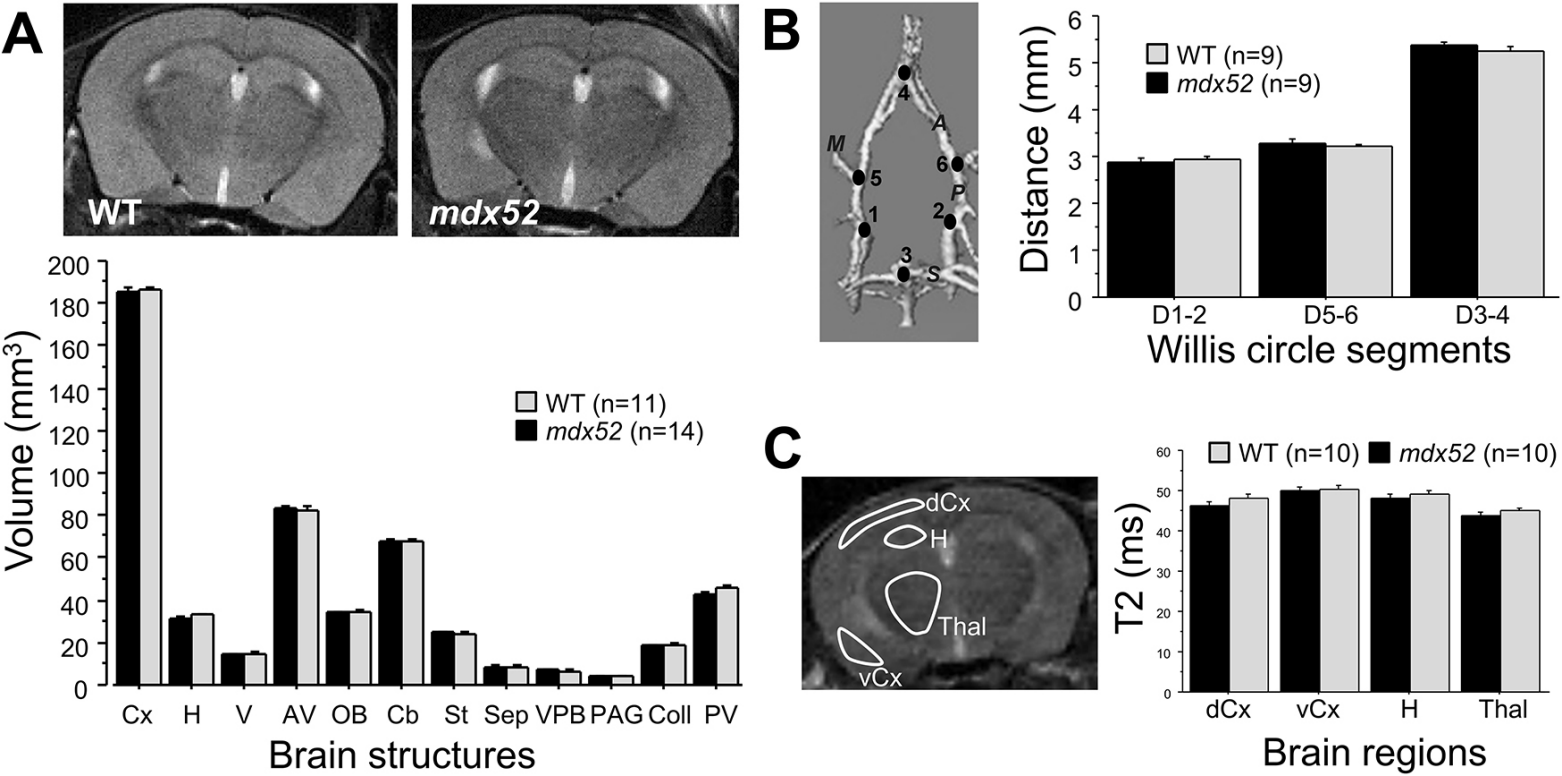
***In vivo* MRI structural analyses.** (A) Top images are representative MRI coronal sections and the histogram below shows the volumes (mm^3^) of the analyzed brain structures, as defined in Table 1. MRI volumetric analyses are shown in the histogram for wild-type (WT, grey bars) and *mdx52* mice (black bars). (B) Angiographic estimation of the length of the main cerebral arteries. The three-dimensional image shows the medial (M), anterior (A), posterior (*P*) and superior (S) arteries, and the six points defined around the Willis circle used to quantify distances (D) between the points (in mm) D1 and D2, D5 and D6, and D3 and D4 in the histogram (right). (C) T2 maps generated at Bregma −1 mm in four brain regions shown in the sample MRI coronal image on the left: dorsal (primary sensory) and ventral (piriform) cortex layers (dCx and vCx, respectively), hippocampus (H) and thalamic nuclei (Thal). T2 quantifications are shown in the histogram (right). Data are mean±s.e.m. Statistics undertaken with two-way ANOVA for repeated measures were all insignificant (*P*>0.05).

We then explored brain neuroanatomy to determine whether the loss of brain dystrophins induced major neurodevelopmental macroscopic brain changes, in particular because Dp140 normally shows highest expression levels in the developing brain compared to the adult brain (Morris et al., 1995). The selection of regions of interest (ROI) was not only driven by functional hypothesis, as putative developmental alterations may affect any region normally expressing Dp427 and Dp140. Hence, we first selected structures showing sufficient contrast to reliably delineate volumes from MRI images on the basis of the mouse brain atlas. The *in vivo* MRI volumetric measurements showed little inter-individual variations within genotypes (Fig. 1A; *n*=11 wild type and 14 *mdx52* mice). The total brain volume was comparable between genotypes [*mdx52*, 521.89±4.86 mm^3^; wild type, 526.75±4.97 mm^3^; genotype effect, *F* (1,23)=0.47, *P*>0.49]. Volumes of the 12 studied brain structures (Table 1) were also comparable between genotypes, whether we analyzed raw mean volumes [genotype×structure interaction, *F* (11,253)=0.45, *P*>0.9] or relative volumes following normalization to the volume of the whole brain [*F* (11,253)=0.81, *P*>0.6].

**Table 1.**
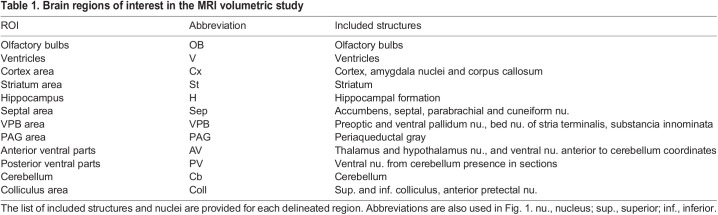
Brain regions of interest in the MRI volumetric study

The absence of gross macroscopic changes was confirmed by comparing the lengths of the main cerebral arteries forming the Willis circle (Fig. 1B), as estimated by angiography [genotype effect, *F* (1,16)=0.56, *P*>0.45; genotype×distance interaction, *F* (2,32)=0.86, *P*>0.42]. Moreover, T2 maps analyses (Fig. 1C) were undertaken in four main regions: we discriminated the primary sensory cortex and the piriform cortex, a prominent cortical area of the rodent brain, important as a main recipient of olfactory inputs and for its widespread projections to forebrain structures, such as the amygdala and the thalamus. We also analyzed the hippocampus, a major structure involved in cognitive functions, and the thalamus, which has extensive connections to the cerebral cortex and midbrain, and plays a major role in relaying motor and sensory signals between subcortical, cerebellar and cortical areas. The T2 maps analyses did not reveal any overall effect of genotype [*F* (1,18)=0.78, *P*>0.38] in the four selected regions [genotype×region interaction: *F* (3,54)=1.15, *P*>0.33], suggesting an absence of major alterations of white-matter tissue microstructure.

**Fig. 2.**
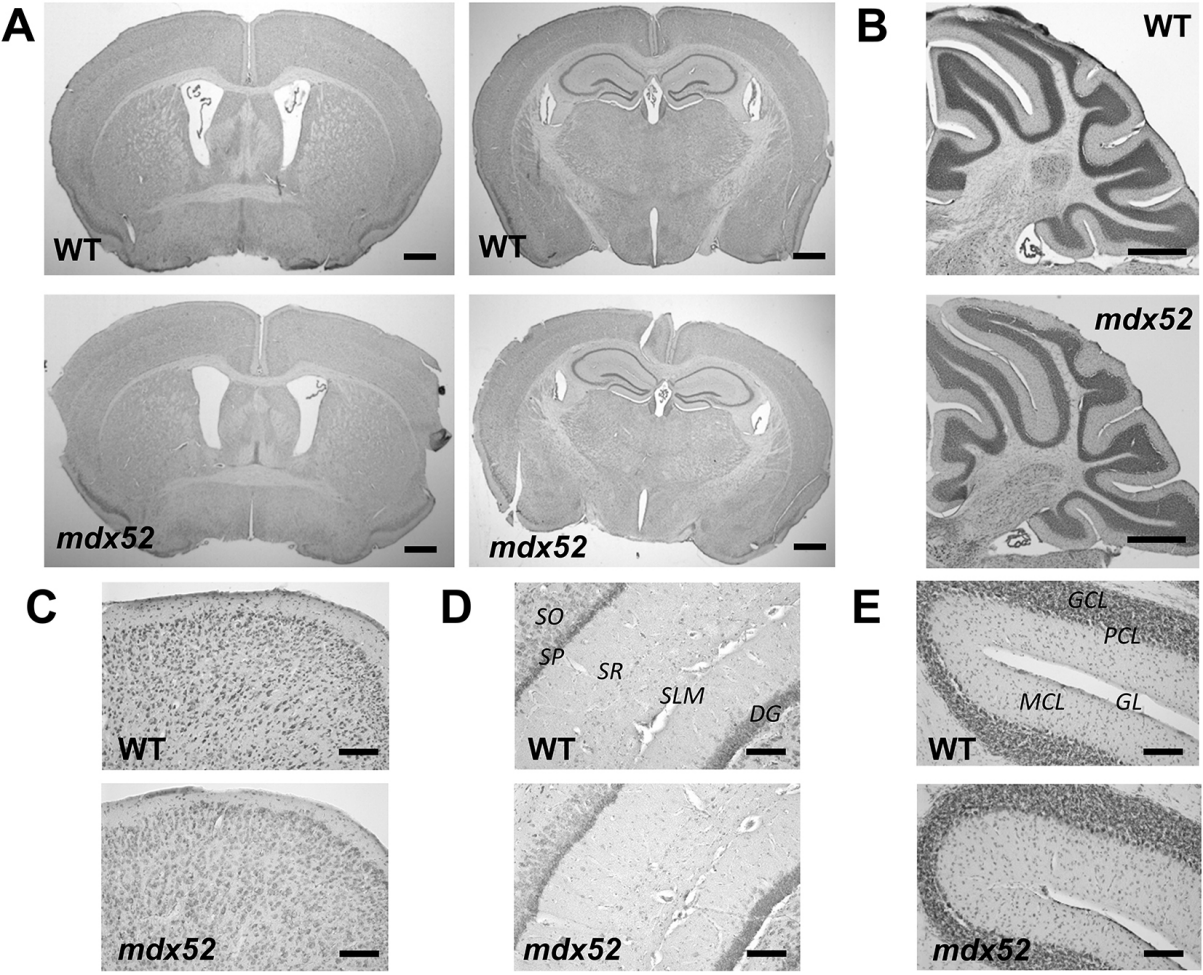
**Brain histology.** (A) Sample light micrographs of Nissl-stained coronal forebrain sections taken at Bregma 0.14 (left) and Bregma −1.7 (right). (B) Sample light micrographs of Nissl-stained sagittal sections (lateral, 0.84 mm), including the cerebellum. (C-E) Images taken at higher magnification to show the general organization of neuronal layers in dorsal cortex (C), dorsal hippocampus (D) and cerebellar lobules (E). DG, dentate gyrus; GCL, granular cell layer; GL, glia limitans; MCL, molecular cell layer; PCL, Purkinje cell layer; SLM, stratum lacunosum moleculare; SO, stratum oriens; SP, stratum pyramidale; SR, stratum radiatum. Scale bars: 500 µm (A,B); 200 µm (C-E).

To further explore general brain architecture and cellular organization, we processed fixed forebrain and cerebellar sections for Nissl staining and histological evaluation. The general appearance and organization of forebrain structures (Fig. 2A), ventricles and fiber tracts (corpus callosum and anterior commissure), as well as of cerebellar lobules and glia limitans (Fig. 2B), were comparable between the genotypes. The brain of *mdx52* mice was properly divided into two hemispheres and the cellular lamination of cortex, hippocampus and cerebellum appeared unaffected at both low (Fig. 2A,B) and high (Fig. 2C-E) magnifications, with no sign of dysplastic cerebral cortex or heterotopic or ectopic cells. In all, there was no overt sign of gross structural malformations or migration defects as reported in some developmental disorders associated with intellectual disability, as for instance in mouse models of congenital muscular dystrophies that hold a deletion of the transmembrane component of dystrophin-associated complex dystroglycan (Satz et al., 2010; Vaillend et al., 2008).

### Behavioral study

#### Exploration and emotional reactivity

**Fig. 3.**
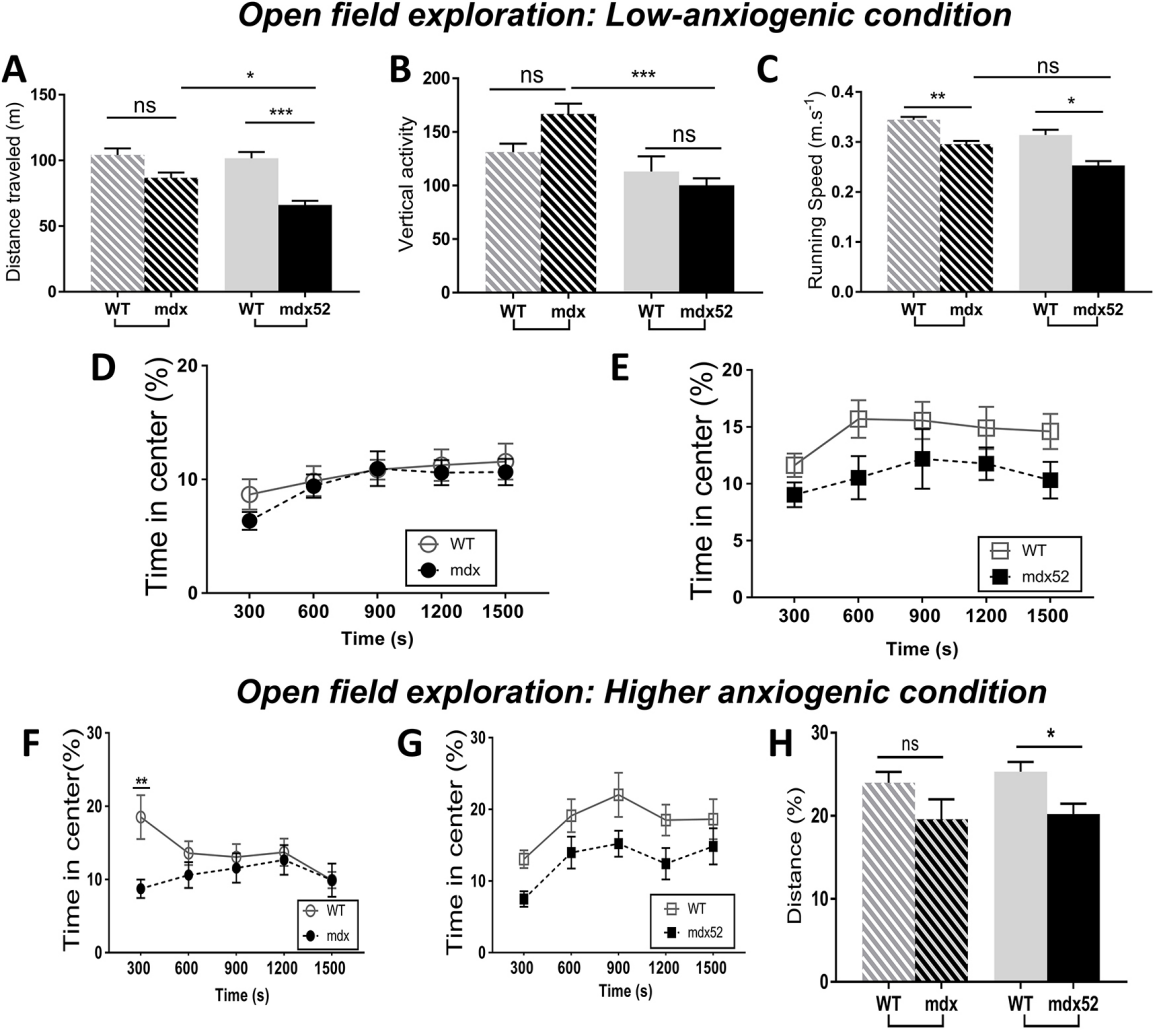
**Exploration of an open field.** (A-E) Exploration during 25 min in a low-anxiogenic condition in 2-month-old *mdx* (*n*=15) mice and their wild-type (WT) littermates (*n*=14), and in *mdx52* mice (*n*=12) and their wild-type littermates (*n*=9). (A) Locomotor activity expressed as the total distance traveled (m). (B) Vertical activity defined by the number of rearings/leanings. (C) Mean running speed (m.s-1). (D,E) Anxiety-related behavioral responses: percentage of time spent in the center zone of the arena expressed by bins of 300 s in *mdx* and wild-type mice (D) and in *mdx52* and wild-type mice (E). (F-H) Exploration of an open field in a more anxiogenic context using distinct cohorts of mice of the same age (*Mdx*, *n*=12 and their wild-type littermates, *n*=13; *Mdx52*, *n*= 15 and their wild-type littermates, *n*=14). Plots show the percentage of time spent in the center zone of the arena by bins of 300 s in *mdx* and wild-type littermates (F), and in *mdx52* mice and wild-type littermates (G). (H) Percentage distance traveled in the center area during the whole session in the four groups of mice. Data are mean±s.e.m. ***P<0.001; **P<0.01; *P<0.05; ns, not significant (four-group comparisons using a Kruskal–Wallis test followed by two-group post-hoc comparisons with Dunn's test). Time courses by 5-min bins analyzed using two-way ANOVA for repeated measures.

Spontaneous locomotor activity was analyzed during exploration of an open field arena for 25 min and compared in *mdx* and *mdx52* mouse models and their respective wild-type littermates (Fig. 3A-E).

The Kruskall–Wallis global analysis revealed significant differences among the four groups for all parameters, and two-group comparisons were further analyzed using post-hoc Dunn's tests. No statistical differences were detected between the two control lines (C57BL/10 and C57BL/6) for main locomotor activity parameters (Fig. 3A-C), reflecting that spontaneous locomotor activity was globally comparable in the two genetic backgrounds. However, we preferred to rigorously compare each mouse model to its wild-type littermate group. No significant differences were detected between *mdx* mice and their wild-type littermates for the mean distance traveled (horizontal activity, Fig. 3A) and vertical activity (Fig. 3B), but the running speed was lower in *mdx* mice (Fig. 3C). In contrast, the *mdx52* mice showed significant reductions for both the distance run and running speed compared to their wild-type littermates, as well as to the *mdx* mice. The large and significant reductions in horizontal activity selectively detected in *mdx52* mice compared to their wild-type littermates suggest that the deletion of exon 52 has a greater impact on locomotion than the original *mdx* mutation.

To evaluate anxiety-related behavioral responses, we analyzed the relative time spent and distance run in the center zone of the open field arena in two different contexts. For the first group of mice described above, open field exploration was undertaken in the presence of sawdust and low illumination, which corresponded to a low-anxiogenic condition. The relative time spent and distance run in the center zone of the open field arena were expressed as a percentage of total time and distance to avoid biased conclusions due to the differences in general activity among groups (Fig. 3D,E). For the time spent in the center, there was a main group effect [*F* (3,47)=4.078; *P*=0012], and post-hoc analyses revealed a significant difference between the two wild-type backgrounds (*P*=0.04), as the C57BL/6 mice spent more time in the central area compared to the C57BL/10 line. There was no statistical difference between *mdx* and *mdx52* mice with this parameter. Comparisons of each mutant group with its respective wild-type littermate control group showed that the percentage of time spent in the center zone by bins of 300 s was equivalent between *mdx* and wild-type littermates (genotype effect, *P*>0.44), and not significantly different between *mdx52* and wild-type littermates (*P*=0.054). However, there were significant differences for the percentage of distance traveled in the center for 25 min (Fig. S2A,B) between *mdx52* mice and their wild-type littermates [*F*(1,20)=4.773; *P*=0.041], but not between *mdx* and wild type [*F*(1,27)=0.5487; *P*>0.4].

To better estimate anxiety during open field exploration, we then tested new groups of mice in a more anxiogenic context, i.e. in the same open field without sawdust and with a higher illumination (100 lx instead of 50 lx). In this condition, the total distance traveled and vertical activity were comparable among groups, whereas reduced running speed was selectively detected in *mdx52* mice compared to their wild-type littermates (Fig. S2C-E). The percentage of time spent in the center zone by bins of 300 s is shown in Fig. 3F,G. Here, the mean difference between the two wild-type lines was not significant (*P*=0.09), but the different time courses were associated with significant differences at specific time points (300, 900 and 1500 s, each *P*<0.02). For *mdx* mice the percentage of time spent in the center zone in this more anxiogenic condition was significantly reduced compared to their wild-type littermates, selectively during the first 5 min of the test [genotype effect, *F* (1,23)=2.898, *P*>0.1; time×genotype effect, *F* (4, 92)=2.856, *P*<0.03; Fig. 3F]. In contrast, in *mdx52* mice, the percentage of time in the center zone was reduced compared to their wild-type littermates during the entire testing period [genotype effect, *F* (1,27)=7.923, *P*=0.009; time×genotype effect, *F* (4, 108)=0.173, *P*>0.9; Fig. 3G). Although both *mdx* and *mdx52* mice showed a tendency for a reduced percentage of distance traveled compared to their respective wild-type littermates, the difference was only significant for *mdx52* mice (Fig. 3H), and comparisons of the distance traveled by bins of 300 s confirmed these conclusions (Fig. S2F,G). Overall, this suggested a transient effect of this anxiogenic context in *mdx* mice, and a persistent effect in *mdx52* mice.

**Fig. 4.**
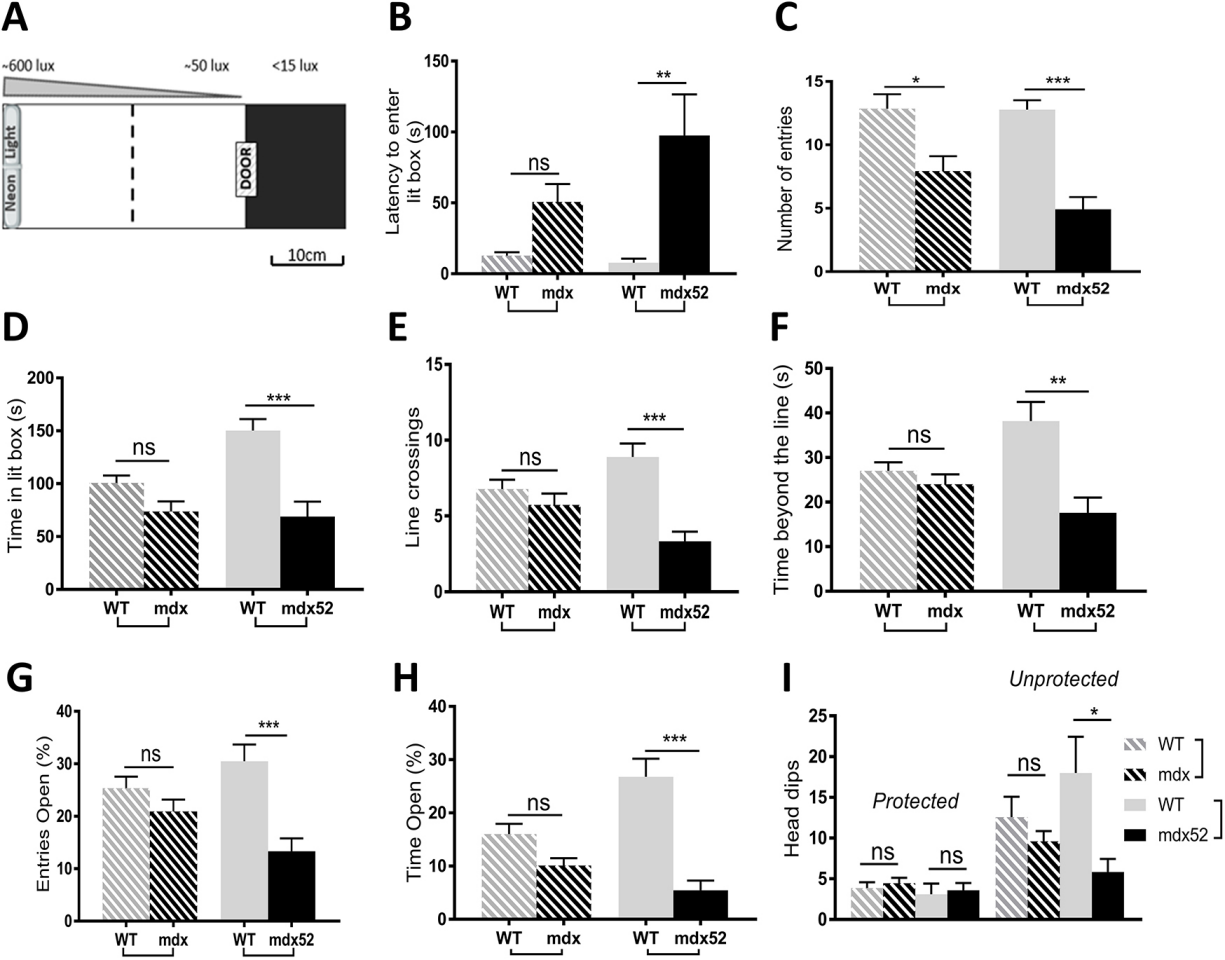
**Anxiety in light-dark choice and elevated plus-maze tests.** Both tests were performed in the same 2-month-old groups of *mdx* mice (*n*=15) and their wild-type (WT) littermates (*n*=14), and in *mdx52* mice (*n*=12) and their wild-type littermates (*n*=9). (A-F) Light-dark choice test. (A) Schematic representation of the light/dark test apparatus (top view) composed of a dark box (15×15 cm), in which each mouse was first introduced, and a lit box (40×15 cm). The two compartments were connected by a sliding door. The lit box was separated in half by a virtual line (dotted line) to analyze behavior in the first and second parts of this box, which had different light intensities; the light intensity gradient is shown above the drawing. The end part of the lit box (600 lx) was considered to be more anxiogenic than the first part near the door (50 lx). Parameters were measured for 5 min. (B) Latency of the first entry into the lit box (s). (C) The number of entries in the lit box. (D) Time spent in the lit box. (E) Number of crossings of the virtual line. (F) Time spent beyond the virtual line, i.e. in the most anxiogenic part of the apparatus. (G-I) Elevated plus maze. Behavioral parameters were analyzed for 5 min. (G) Number of entries in open arms, expressed as percentage of total number of arm entries. (H) Percentage time spent in open arms. (I) Number of head dips made in closed arms (protected) and in open arms (unprotected). Data are mean±s.e.m. ***P<0.001; **P<0.01; *P<0.05; ns, not significant (Kruskal–Wallis test followed by Dunn's test).

The first groups of mice submitted to the low-anxiogenic open field test (Fig. 3A-E) were also tested in two additional anxiety tests: the light-dark choice and elevated plus-maze tests. The light/dark choice, illustrated in Fig. 4A (left panel), is based on the choice given to the mice to stay in a secure dark compartment or to explore an anxiogenic (brightly lit) compartment characterized by a gradient of illumination from the trap door (50 lx) to the end of the compartment (600 lx). As shown in Fig. 4B-D, both mutants display longer latencies and fewer visits compared to their respective wild-type littermates. However, for the latency, only *mdx52* mice displayed a significant difference compared to their wild-type littermates (*P*<0.01), and for the number of entries into the lit compartment the significance was stronger in *mdx52* (*P*<0.001) than in *mdx* mice (*P*<0.05). Moreover, only *mdx52* mice spent significantly less time in the lit compartment (*P*<0.001). The mean duration of visits (total time spent in the lit compartment normalized to the number of visits) was comparable between each group of mouse models and their wild-type littermates (Fig. S3A), indicating no main hypoactivity in the DMD mouse models in this test. To further estimate the level of anxiety in this test, we analyzed the number of entries and time spent in the brightest part of the lit compartment, considered more anxiogenic due to its high illumination (600 lx). We used the virtual line dividing the lit compartment in its middle (broken line in Fig. 4A) to quantify the number of line crossings and the time spent beyond the line (in the brightest part of the lit compartment). These parameters were comparable between *mdx* and wild-type mice (Fig. 4E,F). In contrast, *mdx52* mice showed a significantly reduced number of entries and time spent in this highly lit part (anxiogenic) compared to their wild-type littermates, which further supported the conclusion that *mdx52* mice display more anxiety-like responses compared to *mdx* mice.

In the elevated plus-maze test, anxiety results from the threat induced by the void in the elevated open arms. In this test, the total number of visits to all arms was reduced in *mdx52* mice compared to their wild-type controls (*P*<0.001), which was not observed in *mdx* mice (Fig. S3B). As shown in Fig. 4G,H, the percentage of the number of entries and the percentage of time spent in open arms was comparable between *mdx* and wild type, whereas these measures were largely and significantly reduced in *mdx52* mice (*P*<0.001). All groups performed a comparable number of protected head dips (Fig. 4I), i.e. head dips towards the void while their body is still inside a closed arm. In contrast, only *mdx52* mice showed a reduced number of unprotected head dips (head dips towards the void while being within an open arm; Fig. 4I). However, the number of head dips once normalized to the percentage of time spent in the respective arms showed no more differences between *mdx52* and wild type (Fig. S3C), indicating that the reduced number of unprotected head dips in *mdx52* mice simply reflected their shorter time spent in the more anxiogenic open arms. Overall, the three tests converge towards higher levels of anxiety in *mdx52* mice compared to *mdx* mice and wild-type controls.

#### Unconditioned and conditioned fear responses

**Fig. 5.**
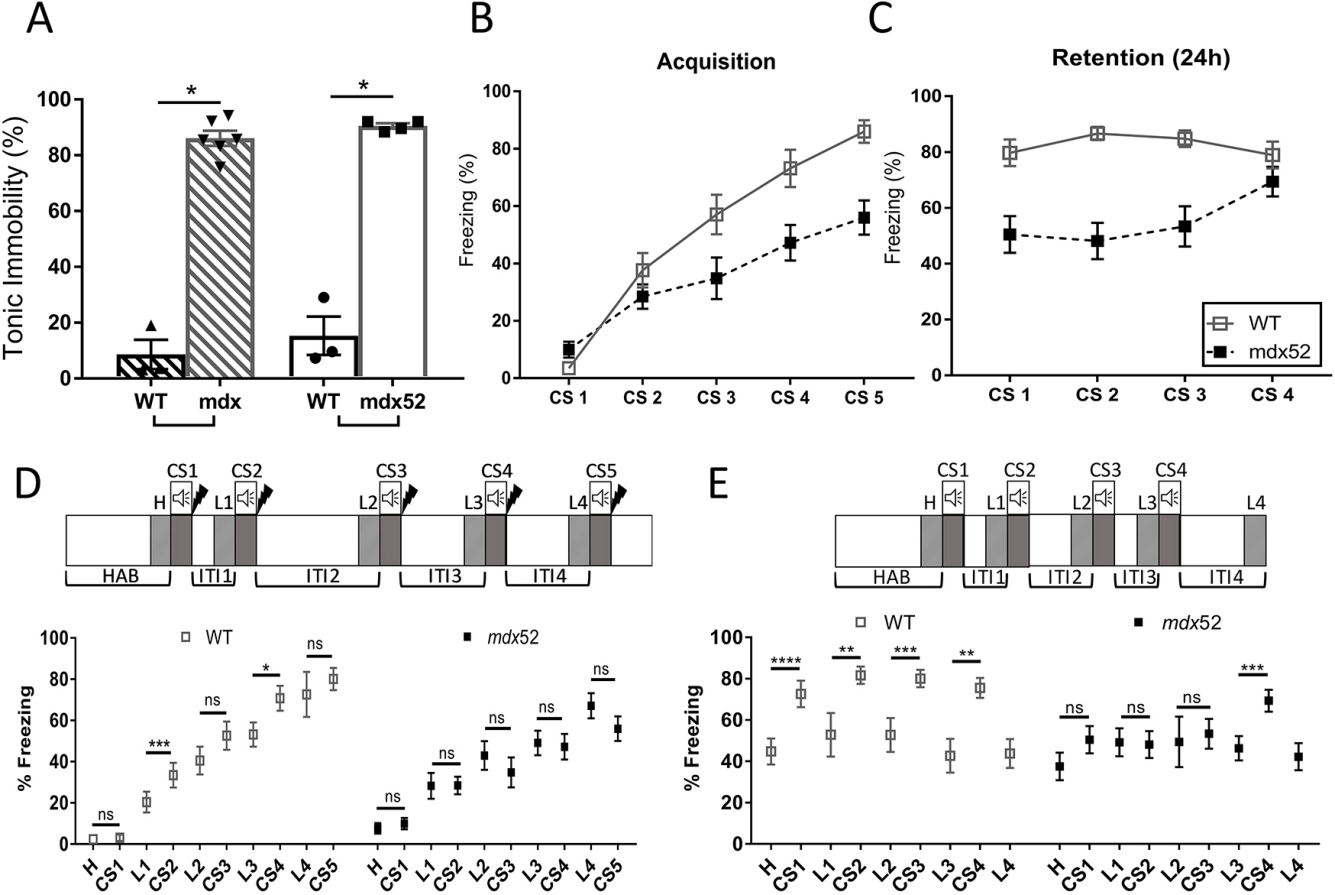
**Fear-related tonic immobility.** (A) Unconditioned fear expressed as the percentage of time spent in tonic immobility (% freezing) during a 5-min period following a brief scruff restraint (15 s). This was compared in 4-month-old *mdx* mice (*n*=6) and their wild-type (WT) littermates (*n*=3), and in 7-month-old *mdx52* mice (*n*=4) and their wild-type littermates (*n*=3). Symbols show individual scores. Statistical significance was determined using a Kruskal–Wallis test followed by Dunn's test. (B-E) Auditory-cued fear conditioning. Fear learning (B, conditioning session) and fear memory (C, retention session) were assessed in adult (3-4 months) *mdx52* mice (*n*=18) and wild-type littermate mice (*n*=16). Learning performance was expressed as the percentage of time spent freezing during presentation of the conditioned stimulus (tone, 30 s), repeated five times during acquisition (CS 1-5; each was an 80 dB tone lasting 30 s, followed by a footshock) and repeated four times (CS1-4) during the retention session performed 24 h later in a new context (fear memory). Genotype differences were significant in both sessions (genotype effects and genotype×trial interactions; two-way ANOVA for repeated measures). (D,E) Detailed analyses of the changes in freezing induced by the CS presentations during acquisition (D) and retention (E) sessions. Diagrams show the experimental sequences: habituation (HAB), successive tone presentations (CS, loud-speaker symbol), shock delivery (lightning symbol) and ITIs of various durations. Freezing during the last 30 s of habituation (H) and last 30 s of ITIs (L1-L4) (hatched areas) were each statistically compared to each following 30-s CS presentation (gray areas) using paired *t*-tests. Plots below show the freezing (%) during each analyzed period in both genotypes (as indicated) and significance of paired comparisons are shown. Data are mean±s.e.m. *P<0.05; **P<0.01; ***P<0.001; *P*<0.001; ns, not significant.

We first studied the stress-induced freezing response, an innate antipredator fear-related behavior, characterized by a complete tonic immobilization of the mouse, except for respiration. It is expressed as the percentage of time spent in tonic immobility (percentage of freezing) during a 5-min period following a brief (15 s) manual scruff restraint, which is considered to be a measure of unconditioned fearfulness. This manual restraint did not result in any observable fear response in the wild-type mice (mean percentage of time spent immobile, <20%). In contrast, the scruff restraint induced a large quantity of freezing in both *mdx* and *mdx52* mice (>80% during the 5 min that followed the stress; Fig. 5A). Even if *mdx* mice in this experiment were younger (4 months old) than *mdx52* mice (7 months old), it has previously been demonstrated that freezing amounts in *mdx* mice reach plateau values at 3 months (Sekiguchi et al., 2009; Vaillend and Chaussenot, 2017). The present results show that, at the adult age, *mdx52* mice display amounts of freezing comparable to that of *mdx* mice.

We then investigated fear learning and memory using auditory-cued fear conditioning, which involves the learning of an aversive cue-outcome association. This test reliably showed delayed acquisition and retention deficits in the original *mdx* mice in many experimental replicates in previous studies (Chaussenot et al., 2015; Vaillend and Chaussenot, 2017a). Therefore, we studied the conditioned fear response only in *mdx52* mice compared to their wild-type littermates, using an identical protocol and experimental conditions (Fig. 5B-E).

Acquisition of fear conditioning (fear learning) was significantly impaired in *mdx52* mice (Fig. 5B), as reflected by a strongly reduced amount of freezing displayed by *mdx52* mice compared to wild type in response to presentation of the conditioned stimuli (CS, tone) [genotype effect, *F* (1,32)=8.612, *P*<0.007; genotype×trial interaction, *F* (4128)=5.989, *P*<0.001]. In contrast, the freezing displayed during the intertrial intervals (ITIs) between presentations of the successive pairs of conditioned (CS)-unconditioned (US) stimuli (i.e. after shock delivery) was comparable in the two genotypes [genotype effect, *F* (1,34)=0.6248; *P*>0.4; genotype×trial interaction, *F* (4136)=1.509, *P*>0.2; not shown], indicating that *mdx52* mice expressed similar fear responses as wild-type mice following delivery of the US (electric footshock). Moreover, fine examination of recordings showed that CS presentation did not induce other types of fear-related escape behaviors in *mdx52* mice (jumps and rearing against walls), indicating that the reduced freezing reflected a more rapid recovery of ambulation compared to wild-type mice, but not a change in fear expression. The selective reduction of freezing during the CS presentation in *mdx52* mice therefore suggests a specific deficit in learning the predictive value of the CS. Twenty-four hours later (retention session, Fig. 5C), the *mdx52* mice also exhibited reduced quantities of freezing during the four presentations of the CS alone (not followed by electric footshocks) [genotype effect, *F* (1,32)=21.57, *P*<0.0001; time×genotype interaction, *F* (3, 96)=4.493, *P*<0.006], suggesting a performance deficit in the recall of fear memory. However, the amount of freezing displayed by both *mdx52* and wild-type mice during this session was not significantly different from their performance at the end of the acquisition session (i.e. during presentation of CS5 during fear learning) [CS5-learning versus CS1-memory; genotype effect, *F* (1,34)=11.52, *P*<0.01; trial effect, *F* (1,34)=1.52, *P*>0.2; genotype×trial interaction, *F* (1,34)=0.03424, *P*>0,8; not shown]. This suggests that the deficit displayed by *mdx52* mice 24 h post-acquisition rather reflects their reduced initial level of learning.

To further detail associative learning performance during the cued fear conditioning, we used paired *t*-tests to compare the amount of freezing during the last 30 s of the habituation period of 2 min and/or the last 30 s of each ITI (L1 to L4) to the following presentations of CS1 to CS5 (30 s duration each). This allowed us to visualize and analyze the changes in freezing specifically induced by the presentation of each 30-s CS, compared to the freezing expressed during the 30 s preceding its presentation (Fig. 5D,E). During the acquisition session (Fig. 5D), the wild-type mice showed clear increases in freezing during CS presentation compared to ‘baseline’ freezing recorded during the preceding 30 s periods (L1-L3), which was statistically significant for CS2 and CS4, until they reached maximal performance of ∼80% of freezing in response to CS5. In marked contrast, there was no increase in freezing in *mdx52* mice upon presentations of the CS during the whole session, suggesting a deficit in learning the CS-US association. The retention session (Fig. 5E), during which CS were presented without any following electric footshock, largely confirmed this conclusion; the wild-type mice systematically displayed an increase in freezing during each CS presentation (CS1-4) compared to the baseline freezing levels preceding CS presentation (all changes were significant), thus attesting solid memory retention of the conditioned CS-US association. In contrast, the *mdx52* mice did not show significant increases in freezing following CS presentations, except during the last CS presentation (CS4). This performance deficit during the retention session likely reflected the initial level of performance of *mdx52* mice, as they showed comparable freezing levels at the end of acquisition and at the start of the retention sessions.

## DISCUSSION

Neuropsychological studies have given greater consideration to the contribution of a central component in the genesis of emotional and neuropsychiatric disturbances in neuromuscular diseases (Colombo et al., 2017; Ricotti et al., 2016). Therefore, investigating the *mdx52* mouse model is important to further understand how the cumulative loss of brain dystrophins impact brain functions. In the present study, we provide the first behavioral investigation of *mdx52* mice lacking brain Dp427 and Dp140, which evidences an exacerbation of anxiety responses and a different pattern of impairment of conditioned fear compared to the original *mdx* model that only lacks Dp427. Moreover, our macroscopic study of brain morphology and architectural organization suggests that alterations related to the cumulative loss of Dp427 and Dp140 likely depend on putative cellular dysfunction rather than major developmental malformation. The behavioral and cognitive deficits that we characterized here are not exhaustive but already confirm the potential of this mouse model for preclinical use, as behavioral tests can easily be implemented as functional outcome measures when evaluating new treatments targeting the CNS.

### Absence of overt gross brain abnormalities

Macroscopic brain abnormalities, such as ventricular enlargement and cortical atrophy in line with grey and white-matter alterations and/or changes in brain metabolism and perfusion, have been reported in some DMD patients, with an apparently higher prevalence when distal mutations affect expression of several dystrophins, particularly Dp140 (Septien et al., 1991; Anderson et al., 2002; Lee et al., 2002; Doorenweerd et al., 2014, 2017). However, brain autopsies and imaging studies still have not led to consistent results, and a possible link between the loss of specific dystrophins and gross and/or ultrastructural brain abnormalities remains uncertain. Structural MRI was also used in DMD mouse models lacking Dp427, which evidenced that Dp427 loss is not associated with major brain volumetric changes (Kogelman et al., 2018; Miranda et al., 2009), which further supported the hypothesis that developmental changes may be due to the loss of other brain dystrophins. In the present study, we addressed this question using *in vivo* MRI and neurohistological analyses in the *mdx52* mouse lacking Dp427 and Dp140. However, *mdx52* mice did not show any overt changes in the whole brain volume or in the volume of several brain structures involved in cognitive functions. Nevertheless, subtle changes in small structures and/or nuclei have been detected in Dp427-deficient *mdx* mice (Kogelman et al., 2018), as well as progressive alterations with aging in longitudinal studies (Bagdatlioglu et al., 2020), which could be further studied in *mdx52* mice in future imaging studies. We cannot exclude that alterations occurring during fetal brain development may have been compensated in the postnatal period, but the present data do not favor major and lasting macroscopic brain malformations in this model.

Dp140 is more highly expressed in the human fetal brain than in the adult brain (Doorenweerd et al., 2017; Lidov et al., 1995; Morris et al., 1995), which suggests it is involved in brain formation, perhaps in neuronal proliferation or migration (Hildyard et al., 2020; Romo-Yáñez et al., 2020). Moreover, the loss of distinct dystrophins reduces the expression of the membrane-spanning core protein of the dystrophin-associated complex, dystroglycan (Kameya et al., 1997; Culligan et al., 2001; Daoud et al., 2009b; Benabdesselam et al., 2012; Belmaati Cherkaoui et al., 2020). Dystroglycan has been implicated in basement membrane formation, cell migration and corticogenesis (Paprocka et al., 2021), as defective glycosylation of the extracellular component of the dystroglycan subcomplex results in defects of the glia limitans and cortical plate disorganization, with overmigration of neurons into the arachnoid space in the Walker–Warburg, Fukuyama congenital muscular dystrophy and muscle-eye-brain syndromes associated with intellectual disability (Godfrey et al., 2007; Guimaraes and Dahmoush, 2020). Although it remains unknown whether dystroglycan interacts with specific dystrophins or utrophin paralogues during fetal brain development, genetic loss of dystroglycan recapitulates the brain abnormalities observed in these congenital muscular dystrophies called dystroglycanopathies (Satz et al., 2010). However, we found no sign of cortical disorganization or modification of the architecture of hippocampus and cerebellum in *mdx52* mice, suggesting that these processes do not require Dp427 and Dp140, or have been compensated by interaction with other brain dystrophins or utrophins.

Overall, the results of this first neuroanatomical study show that the loss of Dp427 and Dp140 is not associated with gross brain abnormalities, similar to what was observed in the original *mdx* mouse that only lacks Dp427. This suggests that the neural dysfunctions resulting from the cumulative loss of these two dystrophins rather take place at the level of brain cellular functionalities. Dp427 has been implicated in GABAergic inhibition and blood-brain barrier functions due to its expression in central inhibitory synapses and vascular smooth muscles, respectively (Belmaati Cherkaoui et al., 2020; Fritschy et al., 2012; Nico et al., 2003). In contrast, the localization and function of Dp140 remain largely unknown. Co-expression networks generated from transcriptomic analyses of human brains suggest putative roles for Dp140 in generic transcription pathways, neuronal differentiation and axonal and dendritic development (Doorenweerd et al., 2017), and experimental studies in animal models revealed expression in vascular and/or glial elements (Caudal et al., 2020; Lidov et al., 1995) and oligodendrocytes (Aranmolate et al., 2017). Oligodendrocytes play a major role in the myelination of neuronal axons, and physical interactions with the vascular endothelium are required for the migration of their precursors (Tsai et al., 2016), which further emphasizes the need for future studies addressing the possible roles of Dp140 in structure connectivity and/or the vascular system.

### Severe disturbances in amygdala-dependent behaviors and associative learning

One of the main challenges for preclinical research aimed at rescuing brain-related dysfunction in DMD is to identify robust behavioral phenotypes in a mouse model with translational value for the clinical condition. The *mdx52* mouse models a frequent genetic profile corresponding to ∼60% of DMD patients (Beroud et al., 2007), which has been primarily associated with emotional behavioral disturbances and cognitive dysfunction (Ricotti et al., 2016). Previous work in *mdx* mice (Sekiguchi et al., 2009; Vaillend and Chaussenot, 2017) has shown that a mild stress, induced by a short (15 s) scruff restraint, results in an exacerbated unconditioned fear response characterized by potent and sustained tonic immobility (>80% of a 5-min time period spent freezing). Here, we found comparable levels of freezing in *mdx* and *mdx52* mice, confirming that this measure of enhanced fearfulness is a reliable outcome measure to evaluate the impact of Dp427 restoration in preclinical studies. This phenotype has been attributed to a dysfunction of the fear circuit, as supported by altered GABAergic inhibitory responses to norepinephrine inputs to pyramidal neurons of the basolateral amygdala in Dp427-deficient *mdx* mice (Sekiguchi et al., 2009).

In this study, we further investigated amygdala-dependent behaviors and learning performance in *mdx* and *mdx52* mice. In the original *mdx* mouse, the presence of anxiety-related responses has been reported, yet the variability among laboratories and the lack of phenotype in the elevated plus-maze test suggested that the changes in anxiety are borderline in *mdx* mice (Manning et al., 2014; Sekiguchi et al., 2009; Vaillend and Chaussenot, 2017). Our present results confirmed this conclusion for *mdx* mice, but further revealed that *mdx52* mice display clearer and significant behavioral changes in all behavioral parameters reflecting anxiety during exploration of the light/dark box, elevated plus maze and open field. It has been reported that muscle wasting may be more severe when mutations affecting expression of muscle Dp427 are expressed in a C57BL/6 background compared with the original C57BL/10 background (Beastrom et al., 2011). Although this may potentially constitute a confounding factor in behavioral analyses, we previously showed in *mdx* mice that reactivity to stress influences locomotor activity, which is otherwise unaltered in unstressed animals (Vaillend and Chaussenot, 2017). In the present study, measures of anxiety were robust in tests with low motor demand, and we normalized anxiety-related parameters to the locomotor activity of an individual to further minimize putative influence of motor factors in our analyses. Besides, the genetic background may also influence brain and behavioral functions (Mortazavi et al., 2021 preprint), which cannot be ruled out without direct comparisons with controls in the same background. Some behavioral parameters were comparable between control mice of the two genetic backgrounds but some others were statistically different, and we therefore preferred to rigorously compare the mutant mice to their respective littermate controls. The significant differences between *mdx52* mice and their littermate controls compared to the non-significant differences between *mdx* mice and their littermate controls suggest that emotional reactivity may be more affected in *mdx52* than in *mdx* mice. This is also in line with the significant deficit shown by *mdx52* mice in the elevated plus maze, which was not observed in *mdx* mice in the present study, as well as in previous reports (Sekiguchi et al., 2009; Vaillend and Chaussenot, 2017). So far, in the present study, we cannot claim a definitive conclusion regarding the severity of the behavioral changes potentially linked to the mutation profile, yet our data show that there are important emotional disturbances in *mdx52* mice that represent robust outcome measures that can be used to evaluate treatment efficacy in future preclinical studies.

Fear conditioning performance was replicated several times by our group in identical experimental conditions in *mdx* mice: the phenotype of this model lacking only Dp427 was consistently characterized by delayed acquisition and retention of the task, but *mdx* mice could finally perform in a comparable manner to their wild-type littermates after repetition of stimulus presentation (Chaussenot et al., 2015; Vaillend and Chaussenot, 2017). In marked contrast, we found here that *mdx52* mice were more severely impaired as they failed to improve their learning performance to reach wild-type performance level and consistently displayed reduced fear responses when auditory CS were presented. However, *mdx52* mice showed similar levels of fear responses as wild-type mice following electric shock delivery during the intervals between CS presentations, indicating that the difference cannot be simply explained by a reduced sensitivity to electric shocks or different expression of fear responses.

To further analyze the cognitive component of fear responses in this task, we compared equivalent time periods just preceding or following CS presentation. Strikingly, this analysis revealed that CS presentation elicited significant increases in fear levels in wild-type mice but not in *mdx52* mice. This was particularly clear during the retention session when no electric shocks were delivered, as the freezing increases observed in wild-type mice were selectively induced in response to CS. In this aversive cue-outcome Pavlovian associative learning, the CS is expected to elicit a specific fear response if animals learn that the CS predicts the delivery of the US (electric footshock). Therefore, our results demonstrate that the performance of *mdx52* mice likely results from the formation of inappropriate or weaker fear associations. This strongly supports a key role for the amygdala in this phenotype, as integrity of this structure is required for fear conditioning (Phillips and LeDoux, 1992). The exact amygdala nuclei and processes remain to be specified. The loss of Dp427 in *mdx* mice likely alters the correct processing of norepinephrine inputs by the basolateral amygdala (BLA) (Sekiguchi et al., 2009). However, the severe phenotype observed here in *mdx52* mice suggests that additional mechanisms involved in the association of fear with a predictive auditory stimulus are affected due to the additional loss of Dp140. This might include alteration of sensory inputs processing, impairment of CS-US association by neurons of the lateral amygdala, altered specificity of the activation of BLA fear neurons by the lateral amyglada and/or impaired plasticity of BLA networks (Herry et al., 2008; Janak and Tye, 2015; Wallace and Rosen, 2001). Although learning the CS-US association was clearly impaired in *mdx52* mice, their reduced performance during the retention session 24 h later does not imply that long-term memory processes, such as consolidation, extinction, reconsolidation and recall processes were impaired. Indeed, the performance of *mdx52* mice during retention was comparable to their level of performance at the end of the acquisition. Hence, it is likely that the retention deficit in *mdx52* mice simply reflected their reduced initial level of learning.

Several animal species with genetic alterations of the *DMD* gene also display strong fear-related behavioral disturbances and enhanced stress reactivity (Frésard et al., 2012; Nonneman et al., 2012; Mori et al., 2015). Moreover, several studies and reports of parents' rating have revealed internalizing problems, including anxiety in Duchenne and Becker muscular dystrophies, which likely contribute to and/or enhance maladaptive social behaviors and neuropsychiatric disturbances (Poysky and Behavior in DMD Study Group, 2007; Young et al., 2008; Snow et al., 2013). According to a recent study of genotype-phenotype relationships (Ricotti et al., 2016), clinical levels of emotional problems are not dependent on the genotype or presence of intellectual disability, and affect a quarter of DMD patients, which represents a high prevalence and supports the face validity of the *mdx52* mouse model characterized by strong emotional and fear-related phenotypes.

In conclusion, in *mdx52* mice lacking Dp427 and Dp140, the disturbances in emotional behavior (anxiety and unconditioned fear) and impairment in learning a Pavlovian CS-US association during fear conditioning appear more severe than in mice lacking Dp427 only. This is in line with the presence of emotional internalizing and externalizing problems, and cognitive deficits in patients with a comparable genetic profile. The behavioral deficits reported in this mouse model confirm that a focus should be placed on the roles of amygdala and brain fear circuit in emotional behaviors, and learning in the context of the central defects associated with DMD. As *mdx52* mice do not display more overt gross brain abnormalities compared to the Dp427-deficient *mdx* mouse, the neural basis of the behavioral and cognitive deficits in this model lacking Dp427 and Dp140 can likely be found at the level of cellular functionalities. Future studies will detail the nature of the deficits and the underlying neurobiological mechanisms resulting from the cumulative loss of these two brain dystrophins. Previous preclinical studies in *mdx* mice revealed that exon-skipping strategies have the potential to alleviate emotional disturbances following treatment at adulthood. The present work provides relevant, robust and translational behavioral readouts to determine the efficacy of such treatments in the *mdx52* mouse model, which recapitulates the most frequent genetic condition associated with brain defects in DMD.

## MATERIALS AND METHODS

### Animals

Exon 52-deleted X chromosome-linked muscular dystrophy mice (*mdx52* mice) were produced by replacement of exon 52 of the *Dmd* gene by the neomycin resistance gene, thereby eliminating expression of Dp427, Dp260 and Dp140 dystrophins but preserving expression of Dp116 (in peripheral nerves) and Dp71 (in brain and retina) (Araki et al., 1997). The mouse line was backcrossed with the C57BL/6J strain for more than eight generations. Breeders were generously provided by Dr. Jun Tanihata and Dr. Shin'ichi Takeda (National Center of Neurology and Psychiatry, Tokyo, Japan). Heterozygous females were crossed with C57BL/6JRj male mice to generate *mdx52* and littermate control (wild type) males in the animal facility of Neuro-PSI at the Université Paris-Saclay in Orsay (France). The C57BL/10ScSn-Dmd*^mdx^*/J (*mdx*) and C57BL/10ScSn lines were originally purchased from Charles River Laboratories, and the breeding of heterozygous *mdx* females with C57BL/10ScSn males generated the *mdx* male mice and wild-type littermates used in this study. All genotypes were determined by PCR analysis of tail DNA*.* Animal care and all experimental procedures complied with the European Communities Council Directive (CEE 86/609/EEC), EU Directive 2010/63/EU, the French National Committee (87/848) and the ethics committee (Paris Centre et Sud, N°59).

### Experimental groups

Only the cages containing mice of both genotypes were selected for experiments. Siblings were kept in groups (two to five per cage) under a 12-h light-dark cycle (light on at 7 am) with food and water *ad libitum*. Behavioral testing was performed blind to the genotype. Mice used for brain imaging, histology and histochemistry were adult males (3-5 months old). For the behavioral study, male mice of the four genotypes (*mdx* and their wild-type littermates, and *mdx52* mice and their wild-type littermates) were tested for exploration and anxiety at the age of 2 months by being successively submitted to three behavioral tests with intervals of 24 h between tests, in the following order: elevated plus maze, light/dark choice test and open field activity. Restraint-induced unconditioned fear was tested in distinct groups of *mdx* and wild-type littermate mice (4 months old), and *mdx52* and wild-type littermates (7 months old). Independent groups of naive mice aged ∼2 months were submitted to a second open field session in a more anxiogenic context. A distinct group of *mdx52* and wild-type littermate mice aged 3-4 months were submitted to the auditory-cued fear conditioning.

### Detection of dystrophins by immunofluorescence

Sagittal sections (30 µm) were cut at −12°C in a cryostat from fresh-frozen dissected brains. Sections were collected on SuperFrost glass slides (Roth, France) and stored at −80°C. For immunochemistry, slides were thawed for 1 min at room temperature, immersed in acetone/methanol (1:1) for 5 min at −20°C, washed three times in 0.1 PBS and incubated in a blocking solution for 45 min [10% normal goat serum (NGS), 0.3% Triton X-100 and 1% bovine serum albumin (BSA)]. Slides were then incubated overnight at 4°C with the following primary antibodies: 5G5 (dilution, neat), a monoclonal antibody directed against the N-terminal part of full-length (Dp427) dystrophin, and the polyclonal H4 antibody (1:800) directed against the C-terminal part of dystrophins and recognizing Dp71 (generous gifts from D. Mornet, INSERM Esprit 25, Université de Montpellier, UFR de Médecine, Montpellier, France). Incubation with primary antibody was followed by washes and incubation with goat anti-mouse IgG or anti-rabbit IgG secondary antibodies conjugated to Cy3 (1:500; Jackson ImmunoResearch, USA, 111-165-003) in PBS (0.1 M) with 5% NGS and 1% BSA for 1 h at room temperature. Cover slips were applied to slides using a mounting medium containing DAPI (Fluoromount-G, Clinisciences, France). No staining was observed in sections processed from control samples from both genotypes when primary antibody was omitted. A laser scanning confocal microscope LSM 700 (Zeiss, France) was used to sequentially collect Cy3 immunoreactivity at 555 nm and DAPI staining at 405 nm. Stacks of 7-15 confocal images and mosaic images (1024×1024 pixels, 16 bits) were imported using an EC Plan-Neofluar 40×/1.30 Oil M27 at a resolution of 156 nm/pixel and processed with ImageJ (National Institutes of Health, USA) using a maximum intensity projection of all the *z*-stack images. All images were randomly taken at the same exposure times and equivalent stereotaxic coordinates.

### Histology and light microscopy

Brains were collected and fixed in a 4% paraformaldehyde fixative solution in 0.1 M phosphate buffer (pH 7.3-7.4) cryoprotected in 30% sucrose and then quickly frozen in 2-methylbutane (Roth, Karlsruhe, Germany) cooled with dry ice to −40°C. Coronal serial sections (30 µm) were collected and stored at −80°C. Nissl stain was performed with thionin and photographed at 1.253× with a Sony DFW-X700 digital camera (Sony, Tokyo, Japan) coupled to an Olympus BX60 light microscope (Olympus, Hamburg, Germany).

### *In vivo* magnetic resonance imaging

Male mice aged 3 months (11 wild-type and 14 *mdx52* mice) were imaged under isoflurane anesthesia (induction 2%; flow rate 1.8-1.2% in 100% compressed air) controlled on the basis of respiratory parameters. The body temperature was maintained at 37°C using a heated mattress. MRI measurements were taken using a 7T horizontal bore magnet (Oxford, UK) driven by Paravision 6.0.1 (Bruker, Wissembourg, France) and equipped with a 300 mT/m actively shielded gradient device (internal diameter, 90 mm, Bruker). For MRI examination, the head of each animal was introduced in a ‘bird-cage’ 1-H coil (internal diameter, 20 mm). After positioning and shimming processes, we performed axial and sagittal anatomic T2-weighted 2D–TurboRARE (rapid acquisition with refocusing echoes) [AXIAL, TR/TE eff=4250/39 ms (repetition time/effective echo time); 8 averages; rare factor=8; 20×20 mm field of view (FOV); 256×256 matrix; 34 slices, 450 µm thickness with 50 µm slice gap; voxel size=78×78×500 µm^3^; SAGITTAL, TR/TE eff=2550/32.6 ms; rare factor=8; lipid removal; ten averages; 20×15 mm FOV; 256×192 matrix; 24 slices, 450 µm thickness with 50 µm slice gap; voxel size=78×78×500 µm^3^] and an angiography [TOF-3D-FLASH sequence (time of flight-fast low angle shot MRI) with TR/Te=14/2.6 ms; excitation pulse angle 20°; 20×20×15 mm FOV; 256×256×96 matrix; voxel size=78 mm^3^]. T2 maps were additionally generated using multi-spin-multi-echo sequence (Tr=2200 ms; 14 echoes time, each 9 ms, first echo time 9 ms; 18×15 mm FOV; 108×96 matrix; pixel size=167×156 µm; seven slices of 800 µm thickness; 250 µm slice gap; one coronal 6-mm saturation slice; time scan 111 s). The total time spent by the mouse in the magnet never exceeded 44 min. After each experiment, mice were recovered from anesthesia and returned to their home cages with free access to food and water.

Areas and volumes were estimated in both the coronal and sagittal MRI planes using AMIRA software (TGS Inc., San Diego, CA, USA). Analyses were performed as described previously (Sebrié et al., 2008) with some modifications: the mouse brain atlas and reference database (Ma et al., 2005; Paxinos and Franklin, 2001) were used as initial guides and the brain was first subdivided into 12 main ROI (Table 1).

Brain volumes were determined by boundary tracing of cross-sectional areas and extracted by multiplying areas by section thickness. Boundary tracing was performed twice by two independent experimenters. MRI angiographic measurements were analyzed by measuring distances between six points defined around the Willis circle (Jamniczky and Hallgrímsson, 2011). Brain T2 maps were generated using PV6.0.1 software (Bruker). Calibration was performed using the signal measured in the tongue muscles at the Bregma level. Intensities for different echo time were measured at Bregma −1 mm and T2 was calculated (R^2^>99% for exponential) following delineation of 4 ROI, including dorsal (dCx) and ventral (vCx) cortical layers corresponding to primary sensory and piriform cortices, respectively, and hippocampus (H) and thalamic nuclei (Thal).

### Behavioral study

#### Elevated plus maze

The maze had two facing arms enclosed with high walls (closed arms, 20×8×25 cm), two open arms without walls (20×8 cm) and a central area (8×8 cm) forming a plus sign situated above a vertical stand to elevate the maze 65 cm above the floor. Illumination was 150 lx in the open and 30 lx in closed arms. Mice were individually placed at the center of the maze with the head facing a closed arm. The number of entries and time spent in open or closed arms were recorded for 5 min. Head dipping over the sides of open arms were counted and classified as protected head dips when the rest of the body of the mouse remained in a closed arm, and as unprotected head dips when the whole body of the mouse was located in an open arm.

#### Light-dark choice

The apparatus had 20-cm-high Plexiglas walls and consisted of a black and dark compartment (15×15 cm; illumination <15 lx) connected by a trap door (6×6 cm) to a brightly lit white compartment (40×15 cm). Bright illumination was provided by a light source placed at the end of the white compartment, opposite from the trap door in order to create an illumination gradient (50 lx close to the trap door to 600 lx close to the light). Each mouse was placed in the dark compartment for 10 s. The trap door was then opened and the mouse was allowed to freely explore the whole apparatus for 5 min. Step through latency, number of entries and total time spent in the lit compartment were manually scored by the experimenter.

#### Open field activity

The test box was a square open field (50×50×50 cm) with black walls and a floor covered with sawdust. Experiments were undertaken under constant room temperature (22–23°C) and homogeneous dim illumination (50 lx). Each mouse was released near the wall and video tracked for 25 min using ANY-maze software (Stoelting, USA). Recorded XY positions were used to generate tracking plots of the exploration paths and to calculate the distance traveled, speed and time spent in distinct zones of the box, i.e. in the whole apparatus and in a central area (30 cm×30 cm, 10 cm from walls), referred to as the center zone. The percentage of time spent and distance traveled in the center zone were used as relative measures of anxiety. The number of rearings and leanings, referred to as vertical activity, were counted manually by the experimenter from saved videos.

In a second experiment with distinct groups of mice, the conditions were modified to increase the anxiogenic context of the test: testing was performed in a higher homogeneous illumination (100 lx instead of 50 lx) and each mouse was released in the center of the white floor of the box, which did not contain sawdust. The floor was cleaned with water then ethanol before each mouse.

#### Restraint-induced unconditioned fear

The mouse was restrained by grasping the scruff and back skin between thumb and index fingers, while securing the tail between the third and little fingers and tilting the animal upside down in order that the ventral part of its body faced the experimenter. After 15 s, the mouse was released to a novel cage (24×19 cm, with 12-cm-high walls) containing clean sawdust and was then video tracked for 5 min under dim illumination (60 lx) using ANY-maze software (Stoelting, USA). Unconditioned fear responses induced by this short acute stress were characterized by periods of tonic immobility (freezing) and quantified during a 5-min recording period. Complete immobilization of the mouse, except for respiration, was regarded as a freezing response (Paylor et al., 2001). The percentage of time spent freezing was calculated for group comparisons.

#### Auditory-cued fear conditioning

The conditioning procedure was carried out using the StartFear system (Panlab S.L., Barcelona, Spain) in the same conditions used previously in our studies of *mdx* mice (Vaillend and Chaussenot, 2017). The conditioning chamber (25×25×25 cm) had three black methacrylate walls, a transparent front door, a grid floor connected to a shock scrambler to deliver US and a speaker mounted on the ceiling to deliver audible tones as CS. The conditioning chamber rested on a high sensitivity weight transducer system to generate an analogical signal reflecting the movement of the animal. The chamber was confined in a ventilated soundproof enclosure (67×53×55 cm) on an anti-vibration table with a surrounding 60-dB white noise. Interchangeable floors and walls (i.e. plain floor and white walls) were used to analyze the retention of cued fear in a novel context. On the first day (acquisition), a 2-min baseline period was recorded before delivery of five CS-US pairs [tone (CS), 80 dB, 10 kHz, 30 s; footshocks (US), each at 0.4 mA for 2 s] with variable and pseudo-randomly distributed intervals between pairs of stimuli (60, 120 and 180 s). On the next day (retention), the session started with the mouse being placed in a different context for 2 min (baseline) before the delivery of four CS (80 dB, 10 kHz, 30 s) separated by intervals of variable durations (60, 90 and 120 s). The movement of the animal was sampled at 50 Hz for quantitative analysis (FREEZING software, Panlab S.L.). Freezing was analyzed during the delivery of the CS (periods of 30 s) to specifically reflect associative learning performance (Chaussenot et al., 2015). *mdx52* mice and their wild-type littermates were gently handled every day for a week before being submitted to tasks, in order to avoid any stress before testing.

### Statistics

Data are presented as mean±s.e.m. Two-tailed statistics were performed using GraphPad Prism7 (San Diego, California, USA). Repeated measures in the MRI and behavioral studies were analyzed using two-way ANOVA with genotype as the between-group factor and temporal variables (brain structure for MRI, and time or trial in behavioral studies). Analyses of performance in cued fear conditioning with ANOVA were followed by detailed comparisons of specific intervals within groups using paired *t*-tests. Four-group comparisons (*mdx* and their wild-type littermates, and *mdx52* and their wild-type littermates) in anxiety tests were analyzed using non-parametric Kruskal–Wallis tests followed by two-group post-hoc comparisons with Dunn's test.

## Supplementary Material

Supplementary information
