# Supplementary material for: Emotional behavior and brain anatomy of the mdx52 mouse model of Duchenne muscular dystrophy
Source: Dis Model Mech. 2021 Sep 21;14(9):dmm049028. doi: 10.1242/dmm.049028 (PMC8476816; doi:10.1242/dmm.049028)
Supplement: Supplementary information [file dmm-14-049028-s1.pdf]

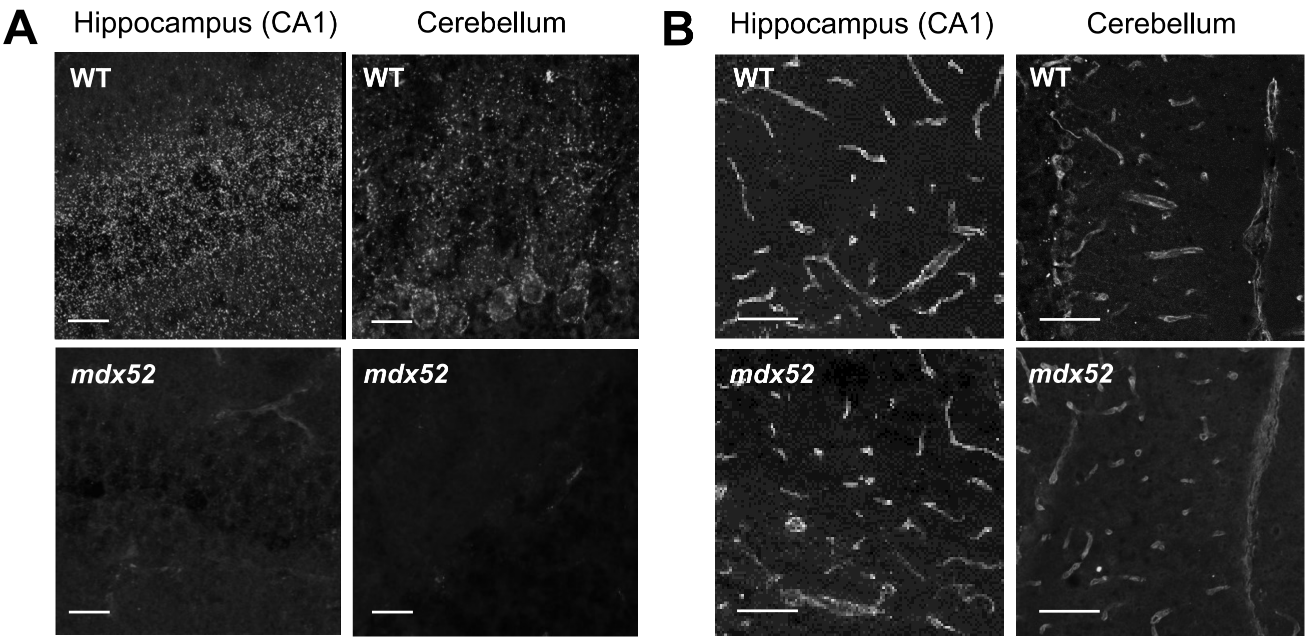

Fig. S1. Detection of brain Dp427 and Dp71 expression in confocal image analyses. **(A)** A monoclonal antibody (5G5) directed against the N-terminal part of Dp427 confirmed its punctate expression in synapses of CA1 hippocampal pyramidal neurons and cerebellar Purkinje neurons in WT mice, and its loss in mdx52 mice. Scale bar: 20 µm. **(B)** A polyclonal antibody (H4) directed against the C-terminal part of dystrophins revealed an immunoreactive signal along walls of blood vessels, which is typical of Dp71 expression in the endfeet of perivascular astrocytes forming the blood-brain barrier. Scale bar: 50 µm.

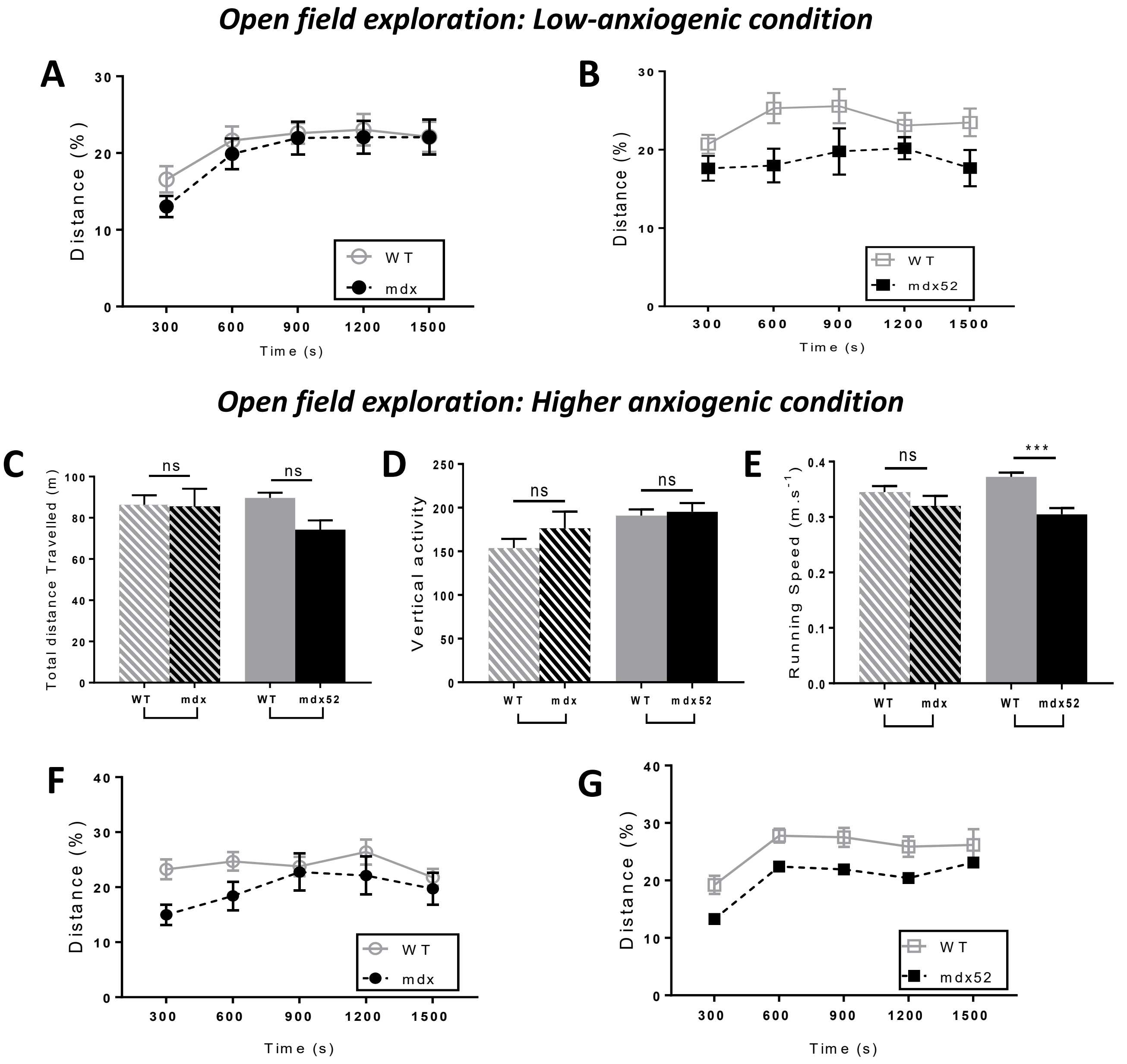

Fig. S2. Additional parameters recorded during exploration of the open field. (A-B) Open-field exploration in the low anxiogenic context: Percent distance travelled in the center area by bins of 300 s during the whole session in mdx and WT mice **(A)** and in mdx52 and WT mice **(B). (C-E)** Behavioral responses in a more anxiogenic context: **(C)** Total distance travelled (m), **(D)** vertical activity, **(E)** running speed (m.s-1). **(F-G)** Percent distance travelled in the center zone by bins of 300s for mdx **(F)** and mdx52 mice **(G)**. ***p < 0.001; ns: p>0.05. Four-group comparisons with Kruskal Wallis test followed by Dunn's test and ANOVAs with repeated measures in A-B and F-G.

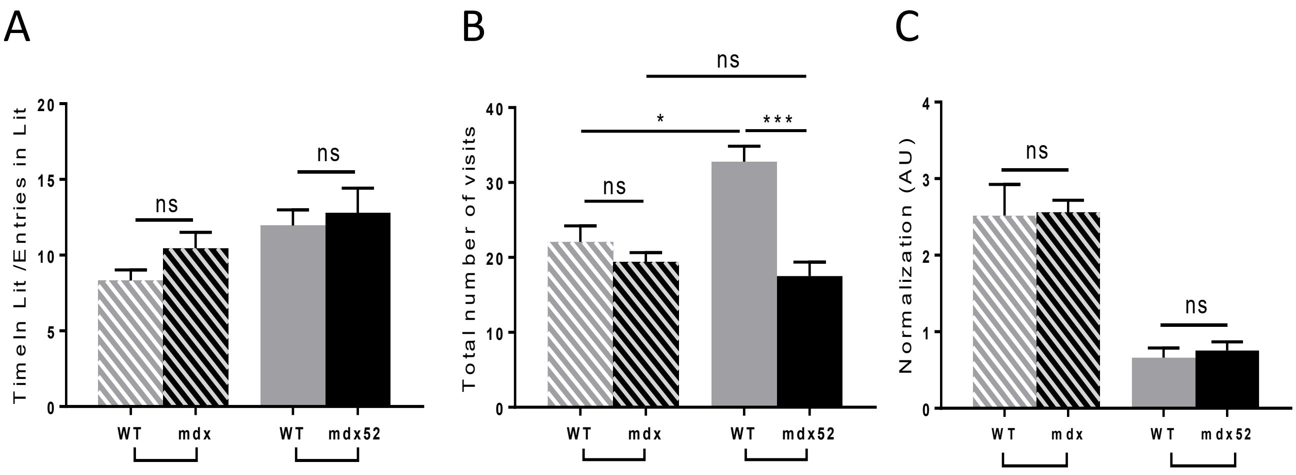

Fig. S3. Additional parameters recorded in light-dark choice and elevated plus-maze tests. (A) Mean duration of visits in the lit compartment in the light/dark choice test. **(B)** Total number of arms visited in the elevated plus maze test. **(C)** Number of unprotected head dips normalized to the percent time spent in open arms. ***$p < 0.001$; *$p < 0.05$; ns: $p>0.05$; Kruskal-Wallis test followed by Dunn's test.
